# Synchronous lung and gastric cancers successfully treated with carboplatin and pemetrexed: a case report

**DOI:** 10.1186/1752-1947-6-266

**Published:** 2012-08-31

**Authors:** Takashi Sato, Koji Tomaru, Tomoko Koide, Makoto Masuda, Masaki Yamamoto, Naoki Miyazawa, Yoshiaki Inayama, Takeshi Kaneko, Yoshiaki Ishigatsubo

**Affiliations:** 1 Department of Respiratory Medicine, Yokohama City University Hospital, 3-9 Fukuura, Kanazawa-ku, Yokohama, 236-0004, Japan; 2 Department of Gastrointestinal Medicine, Yokohama City University Hospital, 3-9 Fukuura, Kanazawa-ku, Yokohama, 236-0004, Japan; 3 Department of Pathology, Yokohama City University Hospital, 3-9 Fukuura, Kanazawa-ku, Yokohama, 236-0004, Japan

## Abstract

**Introduction:**

Lung and gastric cancers are the first and second leading causes of death from cancer worldwide, and are especially prevalent in Eastern Asia. Relatively few reports are available in relation to the treatment and outcome of synchronous lung and gastric cancers, although there are increasing numbers of patients with these cancers. Efforts to develop more effective drugs for the treatment of synchronous cancers, without serious adverse effects, have been intensifying. Pemetrexed, a multi-targeted antifolate enzyme inhibitor, was approved by the United States Food and Drug Administration as a first-line chemotherapy for advanced non-squamous non-small cell lung cancer in 2007. Although clinical activity against several tumor types of adenocarcinoma, including gastric cancer, has been demonstrated, the efficacy of pemetrexed for gastric cancer remains to be fully evaluated.

**Case presentation:**

We report a case involving a 62-year-old Japanese woman with synchronous locally-advanced poorly-differentiated lung adenocarcinoma and poorly-differentiated gastric adenocarcinoma, containing signet-ring cells distinguished by immunohistochemical profiles. She had been treated with carboplatin and pemetrexed as a first-line chemotherapy for lung cancer, and had achieved partial responses for both lung and gastric cancers. These responses led to a favorable 12-month progression-free survival after the initiation of chemotherapy, and the patient is still alive more than 33 months after diagnosis.

**Conclusions:**

This case suggests a new chemotherapeutic regimen for patients with synchronous multiple primary cancers that have an adenocarcinoma background.

## Introduction

The incidence of synchronous cancers has increased because of the reduced mortality resulting from improved treatments and the development of advanced tools for the early detection of cancerous lesions. Although gastric cancer used to be ranked as the most common cancer, especially in Eastern Asia, recent epidemiological studies have revealed that lung cancer is the leading cause of death from cancer in Japan, exceeding gastric, colorectal and breast cancer in rates of mortality [1,2]. Thus, more patients with synchronous and metachronous lung and gastric cancers are now encountered. However, few reports are available regarding treatment and outcome, especially in the case of synchronous forms of these cancers. Here, we report on the case of a patient with synchronous non-small cell lung cancer (NSCLC) and early gastric cancer that were successfully treated with carboplatin plus pemetrexed.

## Case presentation

**Figure 1:**
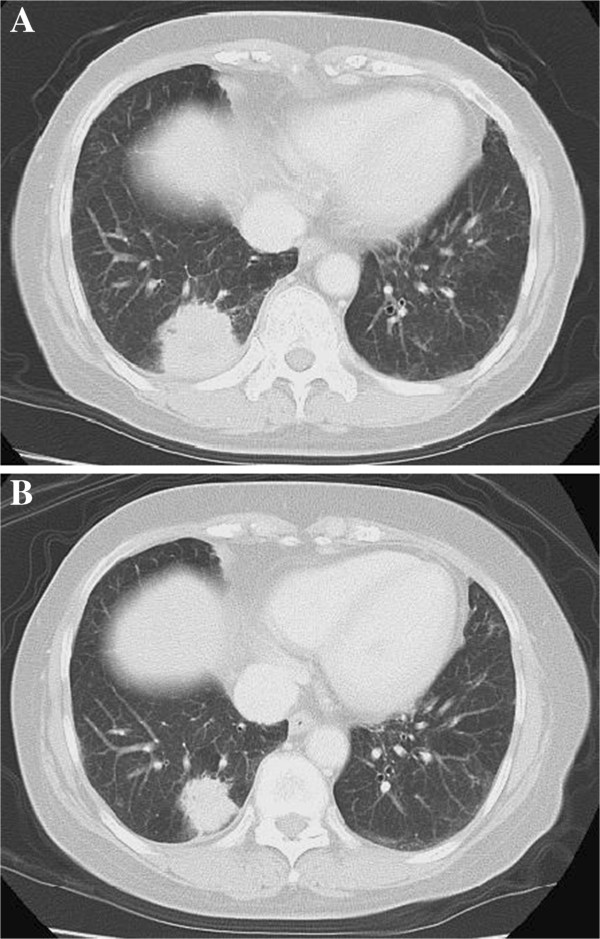
**Gastric lesion observed by gastroscopy before and after treatment with carboplatin plus pemetrexed.** ( **A**) Computed tomography scans before treatment showing the tumor at a maximal diameter of 36mm with an irregular border in segment 10 of the right lung and ( **B**) shrinking tumor judged as a partial response after four cycles of carboplatin plus pemetrexed.

A 62-year-old Japanese woman with a history of smoking presented to a neighborhood clinic because of an abnormal shadow in her right lower lung field seen on a chest X-ray taken during an annual medical checkup. A chest computed tomography (CT) scan demonstrated a solitary lung tumor with an irregular border and a spiculated formation in segment 10 of her right lung, and enlarged right hilar and mediastinal lymph nodes (LNs), suggesting primary lung carcinoma (Figure 1). She was subsequently referred to our hospital for a detailed evaluation.

**Figure 2:**
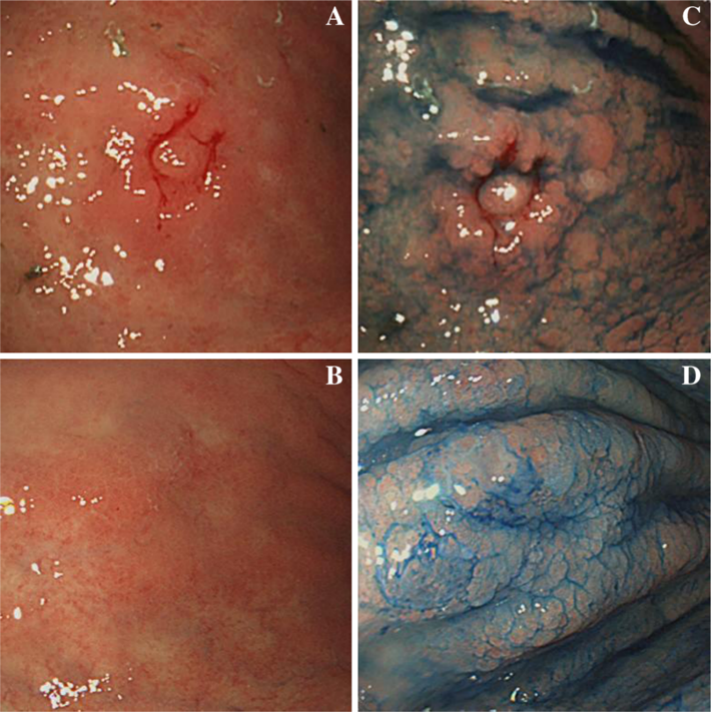
**Gastric lesion observed by gastroscopy before and after treatment with carboplatin plus pemetrexed.** ( **A**) Gastroscopy revealed a slightly elevated mucosal lesion with an irregular surface in the midanterior body of the stomach that was confirmed by biopsy as a gastric adenocarcinoma before treatment. ( **B**) Disappearance of the mucosal lesion after four cycles of carboplatin plus pemetrexed. ( **C**) Chromoendoscopy with acetic acid-indigo carmine showed a mottled image with an indistinct border before treatment and ( **D**) normal mucosa after four cycles of carboplatin plus pemetrexed.

**Figure 3:**
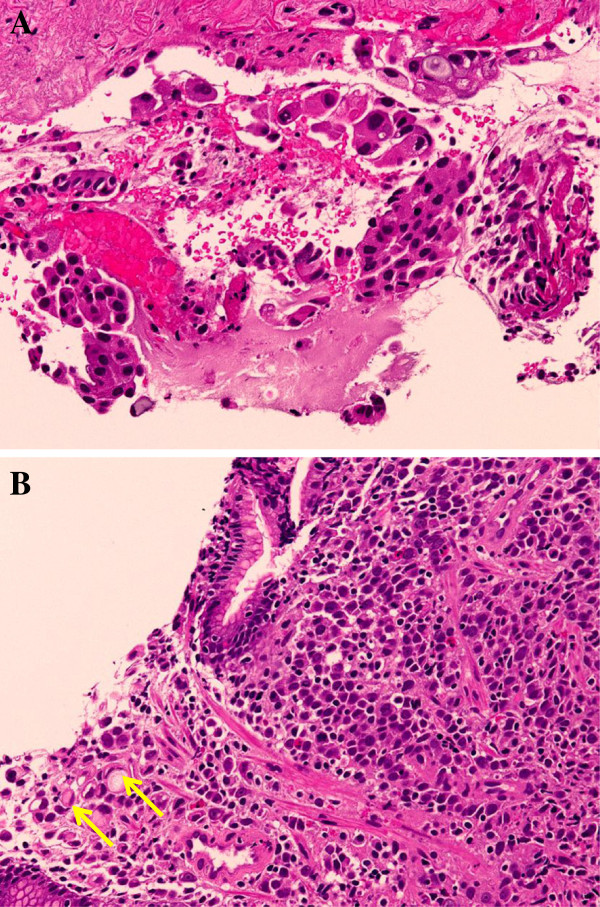
**Pathology of lung and gastric tumors.** ( **A**) Poorly-differentiated papillary adenocarcinoma from segment 10 of the right lung composed of papillary tufts containing fibrovascular cores. Large, atypical cells with enlarged hyperchromatic nuclei were observed (hematoxylin and eosin, original magnification × 200). ( **B**) Tubular adenocarcinoma from the stomach with poorly-cohesive lesions containing signet-ring cells. Discohesive and highly atypical tumor cells with signet-ring cells (arrows) of intracytoplasmic mucin or eosinophilic cytoplasm can be observed (hematoxylin and eosin, original magnification × 200).

Our patient was fairly asymptomatic with no history of respiratory tract infection, chronic cough or bloody sputum; however, she did complain of epigastric pain. Conventional laboratory data showed iron deficiency anemia and elevated levels of carcinoembryonic antigen (121ng/mL) and sialyl Lewis X-i antigen (90μg/mL). Bronchofiberscopy was performed and biopsy specimens were obtained from the primary tumor in segment 10 of her right lung. Microscopic examination of the specimens showed adenocarcinoma without an epidermal growth factor receptor mutation. In addition, gastrointestinal tract endoscopy was performed one day after the bronchofiberscopy, and revealed a gastric cancer located on the antral anterior wall of her stomach (Figure 2). The biopsy specimen was positive for poorly-differentiated adenocarcinoma cells containing signet-ring cells (Figure 3), which were confined within the submucosal layer. Immunohistochemical tests were positive for thyroid transcription factor-1, cytokeratin (CK)7 and mucin (MUC)-1 in the lung tumor, but negative in the gastric tumor. On the other hand, immunostaining was positive for CK7, CK20, MUC5AC and caudal type homeobox 2 expression in the gastric tumor, but negative in the lung tumor, indicating that each adenocarcinoma was a synchronous double cancer. Systemic evaluation, including fluorine-18-fluorodeoxyglucose positron emission tomography, showed no evidence of accumulation other than lung and multiple mediastinal LNs, suggesting a diagnosis of locally advanced NSCLC (Stage IIIA) and early stage IIa + IIb primary gastric cancer. The tumor in her stomach was judged as being an inappropriate case for endoscopic mucosal resection, due to its size and poor histological differentiation.

In accordance with our patient’s request, cisplatin-containing regimens were not considered due to a requirement for massive hydration and hospitalization associated with this type of therapy. For this reason, treatment was initiated with carboplatin (area under the curve: 5) plus pemetrexed (500mg/m^2^) as a first-line chemotherapy for the locally advanced lung cancer on the basis of the available phase III clinical trial evidence [3]. Chemoradiotherapy was not chosen as a treatment option because of the requirement for an extended radiation field. The combination of these drugs was tolerated without any grade 3 or 4 adverse events, determined according to the Common Terminology Criteria for Adverse Events (CTCAE), version 4.0. After four cycles of chemotherapy, the primary lung lesion had been reduced in size and judged as a partial response (PR) using the Response Evaluation Criteria in Solid Tumors criteria (Figure 1). Moreover, the primary gastric lesion was macroscopically diminished (Figure 2), although a few signet-ring cells were still found in biopsy specimens. Along with this favorable outcome, our patient received a further four cycles of maintenance monotherapy with pemetrexed (500mg/m^2^) every three weeks, resulting in stable disease status.

Four months after the last maintenance therapy, chest CT and gastrointestinal tract endoscopy revealed re-growth of the primary lung lesion and recurrence of the mucosal lesion in the stomach, resulting in progressive disease with a 12-month progression-free survival. As a second-line chemotherapy, docetaxel (60mg/m^2^) was initiated and achieved a PR for the lung cancer after three cycles of treatment, although it caused grade 4 leukopenia and neutropenia according to the CTCAE criteria, even after a 20% reduction in dose. After six cycles of treatment, the gastric lesion was evaluated as slowly progressive (still judged as having stable disease status), while the lung lesion was still diminished. A total of nine cycles of docetaxel treatment slowed progressive disease in the case of both the lung cancer in the enlarged mediastinal LNs and the gastric cancer in the extended gastric mucosal lesion. Following chemotherapy with weekly irinotecan (100mg/m^2^) as a third-line chemotherapy, our patient also exhibited grade 4 leukopenia and neutropenia. However, the treatment was successfully administered in the case of the lung cancer, with reduced mediastinal LNs, and was judged as a PR after four cycles of treatment. However, there was no response to this chemotherapy regimen in the case of the gastric cancer, with the disease showing further progression. Our patient is still alive 33 months after diagnosis, and further chemotherapy is planned including oral fluoropyrimidine S-1 as a gastric cancer-oriented therapy.

## Discussion

The incidence of synchronous cancers of the lung and stomach has been reported as being 0.71% and 0.72% in Korean and Japanese patients, respectively [4,5]. Of these patients, about 50% received only radiotherapy or supportive care, mainly due to the advanced stage of the lung cancer, resulting in one- and two-year survival rates of 50% and 32.4%, respectively [5]. Pemetrexed is a recently developed antifolate drug shown to prolong survival time in patients with non-squamous NSCLC and to have a favorable toxicity profile, and was tolerated even in patients who had received it as a third- or further-line treatment [6]. Although activity of pemetrexed against several breast, gastric, pancreatic and colorectal adenocarcinoma cell lines has been reported [7], only a limited number of phase I or II studies involving chemotherapy including pemetrexed have been carried out in patients with gastric cancer; response rates ranged from 23% to 36% [8,9]. In Japan, there is only one case report demonstrating the efficacy of carboplatin plus pemetrexed in patients with gastric cancer [10]. Our case showed the efficacy of docetaxel or irinotecan monotherapy in non-squamous NSCLC, but not in gastric adenocarcinoma. Taken together, we may consider that pemetrexed plus carboplatin has greater potential efficacy and lower adverse effects compared with docetaxel or irinotecan monotherapy in the treatment of advanced gastric cancer, even when it is poorly differentiated.

## Conclusions

Findings from our case suggest a new chemotherapeutic regimen with low adverse effects for patients with synchronous multiple primary cancers with an adenocarcinoma background.

## Consent

Written informed consent was obtained from the patient for publication of this case report and any accompanying images. A copy of the written consent is available for review by the Editor-in-Chief of this journal.

## Competing interests

The authors declare that they have no competing interests.

## Authors’ contribution

TS and TKo analyzed and interpreted the patient data and wrote the manuscript. KT, MM, MY, NM, YIn, TKa and YIs analyzed and interpreted the patient data. All authors have read and approved the final manuscript.
